# Tryptophan Attenuates the Effects of OTA on Intestinal Morphology and Local IgA/IgY Production in Broiler Chicks

**DOI:** 10.3390/toxins13010005

**Published:** 2020-12-23

**Authors:** Fernando Galdino Ricci, Leticia Rodrigues Terkelli, Emerson José Venancio, Larissa Justino, Beatriz Queiroz dos Santos, Ana Angelita Sampaio Baptista, Alexandre Oba, Bianca Dorana de Oliveira Souza, Ana Paula Frederico Rodrigues Loureiro Bracarense, Elisa Yoko Hirooka, Eiko Nakagawa Itano

**Affiliations:** 1 Department of Pathological Sciences, State University of Londrina, P.O. Box 10.011, Londrina, PR 86057-970, Brazil; fernandogricci@hotmail.com (F.G.R.); letterkelli@gmail.com (L.R.T.); emersonj@uel.br (E.J.V.); bianca_dorana@hotmail.com (B.D.d.O.S.); 2 Department of Preventive Veterinary Medicine, State University of Londrina, P.O. Box 10.011, Londrina, PR 86057-970, Brazil; larissa.justino@uel.br (L.J.); beatriz.queiroz@uel.br (B.Q.d.S.); anaangelita@uel.br (A.A.S.B.); 3 Department of Zootechny, State University Londrina, P.O. Box 10.011, Londrina, PR 86057-970, Brazil; oba@uel.br; 4 Laboratory of Animal Pathology, State University of Londrina, P.O. Box 10.011, Londrina, PR 86057-970, Brazil; anapaula@uel.br; 5 Department of Food Science and Technology, State University Londrina, P.O. Box 10.011, Londrina, PR 86057-970, Brazil; elisahirooka@hotmail.com

**Keywords:** IgA, IgY, mycotoxins, villi, crypts

## Abstract

Ochratoxin A (OTA) is a mycotoxin produced by species of *Penicillium* and *Aspergillus* that can contaminate products of plant origin that are used as animal feed. Through oral exposure, this mycotoxin primarily affects the chicken gastrointestinal system. The present study evaluated the intestinal toxic effects of OTA and the introduction of L-tryptophan to alleviate these effects in chickens. One-day-old chicks were exposed to a single OTA dose (1.4 mg/kg body weight—b.w.) and treated with or without four daily doses of L-tryptophan (100 mg/kg b.w.). Duodenal villus height/crypt depth, fecal immunoglobulin A/immunoglobulin Y (IgA/IgY) levels, and duodenal positive immunoglobulin A cells (IgA^+^) were evaluated by histology, ELISA, and immunohistochemistry, respectively, on the 14th day. There were significant changes in the duodenal villus height, crypt depth, and levels of fecal IgA/IgY and duodenal IgA^+^ cells (*p* < 0.05) in groups exposed to OTA. On the other hand, groups exposed to OTA and treated with L-tryptophan showed similar levels of villus height, IgA/IgY levels, and duodenal IgA^+^ cells to those of the control group (*p* > 0.05). In conclusion, exposure to a single dose of OTA orally induces changes in intestinal morphology, levels of IgA/IgY antibodies, and IgA^+^ cells. Thus, treatment with L-tryptophan may be a valid alternative means to reduce the harmful effects of OTA on the intestinal mucosa, which requires further study.

## 1. Introduction

Ochratoxin A (OTA) is a mycotoxin produced by several species of the genus *Penicillium* and *Aspergillus* [1,2,3]. The growth of these fungi, particularly in plant products is favored by climatic conditions such as temperature and humidity. Some of these products are used as ingredients in poultry feed, and when contaminated by OTA, they can cause animal intoxication [4,5]. The ingestion of OTA contaminated feed by poultry causes losses in production, with increased mortality, decreased weight gain, and increased feed conversion, in addition to decreased production and quality of eggs, causing severe economic losses to the productive sector [6,7,8,9].

OTA is one of the mycotoxins with many harmful effects on animals, such as mutagenic, teratogenic, immunogenic, nephrotoxic, and hepatotoxic, in a dose-dependent relationship [8,10,11,12]. OTA is classified by the International Agency for Research on Cancer in Group 2B [13] and for the European Union, the recommendable OTA limit is 0.1 mg/kg in complementary ingredients for poultry feed [14].

OTA is a mycotoxin with high thermoresistance and structurally consists of a dihydroisocoumarin moiety linked with a phenylalanine through an amide bond. Its molecular weight is 403.82, and it is considered a weak organic acid, with a crystalline, solid, transparent, and odorless structure [1,2,3]. This mycotoxin is quickly absorbed by the small intestine in birds [15,16]. OTA affects the gastrointestinal tract (GIT) through inflammatory response/oxidative stress and by decreasing villus height [7,17]. The villus height is important for increasing the intestinal absorption surface and represents an important factor in determining animal performance [18].

The GIT also presents an important protective function against the invasion of microorganisms [18]; however, in this context, there are few studies on the effects of OTA in poultry intestinal mucosa. Increased inflammatory cytokines and decreased anti-inflammatory cytokines due to OTA have been observed in cecal tissues of broiler chickens [17]. Solcan et al. [19] showed a significant reduction in the two main lymphocyte subpopulations, CD4^+^ and CD8^+^, which are cells that are important for the cellular immune response in the intestinal tissues in chickens, depending on OTA dose and exposure time.

One of the important mechanisms in the defense of the intestinal mucosa is the action of antibodies, mainly the noninflammatory IgA. This immunoglobulin is important not only to confine bacteria in the intestinal lumen but also to shape the overall composition of the intestinal microbiota [20]. However, there are no data in the literature on the effect of OTA on local antibody production in intestinal mucosa.

Tryptophan is a nutritionally essential amino acid for humans, and many species of animals and a diet enriched with this amino acid can affect intestinal epithelial cells by inducing anti-inflammatory cytokines [21], as well as enhancing the immune system defense by increasing blood immunoglobulin levels [21]. In broiler chickens, supplementation with tryptophan improved the villus height: crypt depth ratio in basal or low crude protein diet models [22]. A recent investigation showed that treatment with 5-hydroxytryptophan (5-HTP), a tryptophan precursor, increased IgA concentration in the duodenum and ileum of chickens [23].

The current study investigated the effects of a single oral exposure to OTA on duodenal intestinal histomorphology, local IgA/IgY levels, and local positive IgA cells (IgA^+^) in broiler chickens and introduced an L-tryptophan treatment to verify whether it can mitigate the possible toxic effect of OTA. This is the first study to show the effects of OTA on IgA/IgY levels in chicken intestines and that treatment with L-tryptophan reduces its harmful effects.

## 2. Results

### 2.1. Intestinal Morphometry

**Figure 1:**
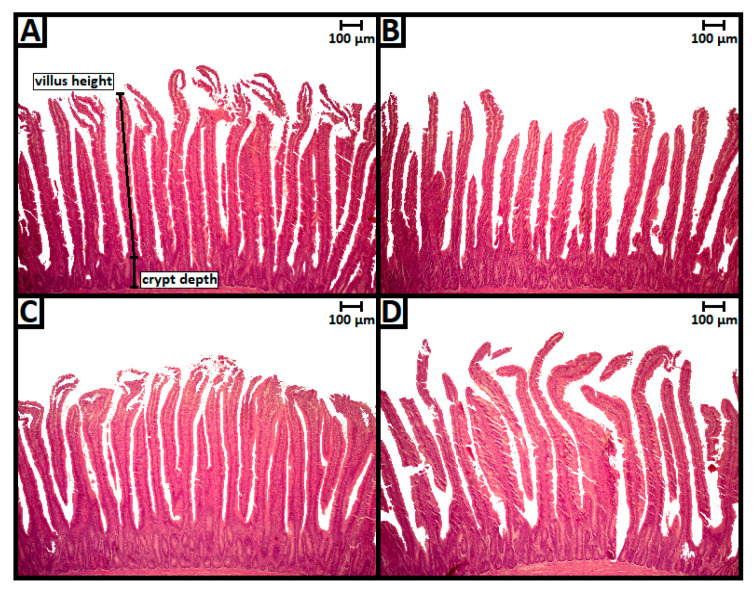
Villi and crypts in fourteen-day-old chickens exposed to ochratoxin A (OTA) at one day of age, in hematoxylin/eosin stain (40× magnification). (**A**) negative control, (**B**) OTA, (**C**) OTA + L-tryptophan, (**D**) L-tryptophan control.

**Figure 2:**
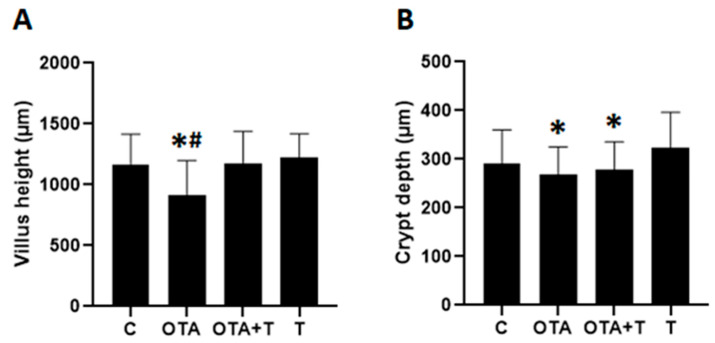
Villus height (**A**) and crypt depth (**B**) in fourteen-day-old chickens exposed to OTA at one day of age. C = negative control, OTA = exposed to single oral OTA dose, OTA + T = exposed to single oral OTA dose and treated with L-tryptophan, T = L-tryptophan control, treated only with L-tryptophan without OTA exposure. * significant reduction when compared to C or T (*p* < 0.05), # difference when compared OTA and OTA + T (*p* < 0.05). Statistical analysis: One-way analysis of variance (ANOVA), Kruskal–Wallis and Student–Newman–Keuls for multiple comparisons.

Histological analysis of the duodenum in chicks exposed to a single dose of OTA orally, at one day of age, resulted in morphological alterations on the 14th day (Figure 1). The villus height and crypt depth of the OTA group decreased significantly in relation to the other groups (*p* < 0.05), (Figure 2). As expected, treatment with L-tryptophan in one of the groups exposed to OTA resulted in a similar villus height to that of the control group and different from the group exposed only to OTA, suggesting the neutralizing capacity of L-tryptophan (Figure 2).

### 2.2. Total IgA and IgY Levels in Fecal Content

**Figure 3:**
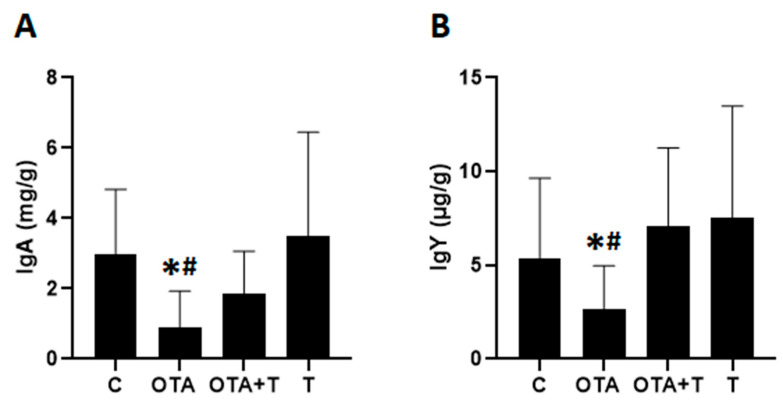
Fecal immunoglobulin A (IgA) (**A**) and immunoglobulin Y (IgY) (**B**) levels in fourteen-day-old chickens exposed to OTA at one day of age. C = negative control, OTA = exposed to single oral OTA dose, OTA + T = exposed to single oral OTA dose and treated with L-tryptophan, T = L-tryptophan control, treated only with L-tryptophan without OTA exposition. * significant reduction when compared to C or T (*p* < 0.05), # difference when compared OTA and OTA + T (*p* < 0.05). Statistical analysis: One-way analysis of variance (ANOVA) and Student–Newman–Keuls for multiple comparisons.

The fecal IgA (Figure 3A) or IgY (Figure 3B) levels decreased significantly in the group of animals exposed only to OTA in relation to the other groups (*p* < 0.05, Figure 3). No significant reduction was observed when the group exposed to OTA (OTA) was treated with L-tryptophan (OTA + T) (*p* > 0.05), suggesting that treatment with L-tryptophan may counteract the toxic effect of OTA.

### 2.3. Immunohistochemistry for IgA Detection in the Duodenum

**Figure 4:**
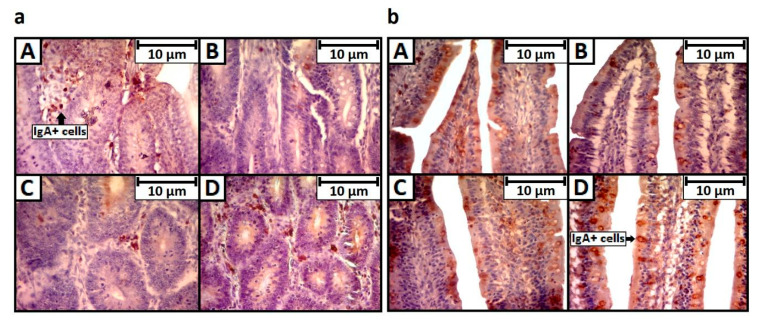
Crypts (**a**) and villi (**b**) in fourteen-day-old chickens exposed to OTA at one day of age, marked for IgA by immunohistochemistry, counterstained by Harris hematoxylin (400× magnification). A: negative control, B: OTA, C: OTA + L-tryptophan, D: L-tryptophan control.

Through immunohistochemistry, fewer points and with less intensity of IgA labeling were observed in the group treated with OTA, both in crypts (a) or villi (b), which is apparently different from the negative control or the group treated only with tryptophan (Figure 4).

### 2.4. Quantity of IgA^+^ Cells in the Duodenum

**Figure 5:**
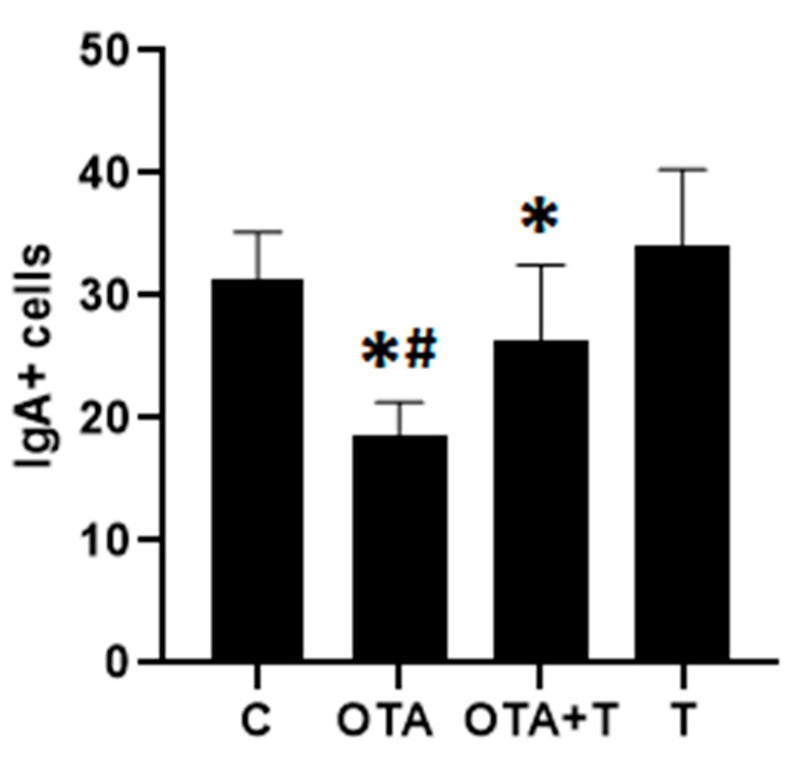
Number of IgA^+^ cells in the duodenum in fourteen-day-old chickens exposed to OTA at one day of age. C = negative control, OTA = exposed to single oral OTA dose, OTA + T = exposed to single oral OTA dose and treated with L-tryptophan, T = L-tryptophan control, treated only with L-tryptophan without OTA exposure. * significant reduction when compared to C or T (*p* < 0.05), # difference when compared OTA and OTA + T (*p* < 0.05). Statistical analysis: One-way analysis of variance (ANOVA) and Student–Newman–Keuls for multiple comparisons OTA + T.

The number of IgA^+^ cells decreased significantly in the OTA group in relation to the other groups (*p* < 0.05, Figure 5). There was also an increase in the means of the groups treated with L-tryptophan compared to the groups not treated with L-tryptophan.

## 3. Discussion

The effects of chronic exposure to a low dose of OTA may have more impact than acute exposure [3]; however, in the present study, a single oral dose of OTA (1.4 mg/kg body weight—b.w.) in newly hatched birds induced morphological alterations in the intestinal mucosa. This dose represents an occasional dose [24], but it is above the recommended maximum daily intake of OTA for poultry feeds by ECR2006/575/EC [14].

A preliminary investigation with 0.1, 0.7, and 1.4 mg OTA/kg b.w. (in one-day-old chickens), decreased leukocyte level was detected only in highest dose (data not shown). Therefore, this high dose, that may eventually occur, was chosen for this study.

Although alterations in intestinal morphologies have been observed with lower OTA doses, these have been after multiple doses in a chronic form, over a long period [19], which can also generate an accumulated high dose.

The intestinal villi height and crypt depth parameter is used as an important indication of gut health in pigs [25]. In the current work, decreased intestinal villus height was detected, which is in agreement with the results observed by Solcan et al. [19], using multiple doses of OTA exposure in the feed.

As the nutrient absorption area is increased by the intestinal villi, changes in the area can negatively affect the absorption of nutrients [24], which may have more impact in young chicks where nutrients are an important factor for the development of the small intestine [26]. Peraica et al. [27] observed a decrease in glucose absorption induced by OTA via the SGLT1 transporter using an in vitro assay.

Herein, the permeability of intestinal mucosa was not investigated. However, in vitro investigations using cellular lines suggest that OTA increased permeability [28], which was most likely by removing specific claudin isoforms from the tight junctions (TJ) that form the gut barrier [29]. The TJ between intestinal epithelial cells maintain a barrier to prevent the entry of toxic substances or pathogens when present in the lumen of the intestinal mucosa.

In addition to the mechanical barrier, the intestinal mucosa has active defense mechanisms and the activation of B lymphocytes and production of IgA are the main defense mechanisms against local pathogens [30,31]. In the present study, a significant reduction in the levels of fecal IgY and IgA and a decrease in the number of IgA^+^ cells in the intestinal mucosa were observed. These results suggest that the local IgA production is suppressed in chickens orally exposed to a single dose of OTA.

A minor source of IgA in secretions is derived from blood via the hepatobiliary IgA transport system [30], and according to Bar-Shira et al. [32], maternal IgA is present in the intestinal mucosa, but it is replaced by its own production at 7–10 days of age in domestic fowls. Therefore, herein, the interference of maternal IgA is possibly negligible at 14 days. In addition, IgA^+^ numbers were lower in duodenal tissues in animals exposed to OTA, suggesting that local IgA production is affected.

Although the histological/immunohistochemical evaluation was restricted to the duodenum, we believe that the same occurred along the extension of the intestine due to the age of the chicks and high OTA dose. However, the antibodies evaluated here, in the cecal content, represent the result of general production by all the intestinal mucosa.

The harmful effects of OTA on intestinal mucosa observed in the current study with a single dose may be due to: (a) the high dose used considering the primary local absorption (40% is passively absorbed in the upper part of the GIT), throughout the non-absorbed toxins and also by enterohepatic circulation, increasing exposure of the entire intestine [24]; (b) chick age, as newly hatched chicks do not yet have a fully developed small intestine [26]; (c) exposure route used, by gavage, which could contribute to greater OTA exposure at the GIT. According to [15,16], absorption can be decreased if it occurs together with feed consumption.

The data obtained here show that even after a single dose, if consumed at the beginning of life, some of the harmful effects can last for at least 14 days, and possibly longer, which requires further study.

Interestingly, the results showed that the oral administration of L-tryptophan improves the intestinal villi height in chickens exposed to OTA, suggesting that L-tryptophan treatment can neutralize or reverse OTA action, therefore improving the health of the intestinal mucosa. This is in accordance with Nakamura et al. [33], who evidenced the development of microvilli in the intestine by both endogenous and exogenous 5-HTP, which is a tryptophan precursor.

Another important data in this work was the evidence that the tryptophan treatment can neutralize or mitigate the suppressive effect of OTA on IgA production by the chicken intestine. This beneficial effect could also be by the direct action of the serotonin, a tryptophan derivative, contributing to the improvement in the immune response. Serotonin, derived from 5-HTP, presents immunomodulatory activity on immune cells [34,35,36] and can also upregulate the proliferation of B lymphocyte stimulated with mitogen through 5-HT_1A_ receptors [37]. In addition, serotonin has scavenger or anti-oxidant activity, and one of the toxic effects of OTA is the induction of oxidative stress, leading to cell death [35]. Thus, it could be that direct action and also the inhibition of antibody-producing cells or even death of their precursor cells could have contributed to the normalization of IgA levels through tryptophan treatment in OTA-intoxicated chickens.

By using another type of tryptophan, N-acetyl-L-tryptophan (NAT), Argawal et al. [38] evidenced a protective action against the effects of OTA, such as an inhibition of cell proliferation, prevention of protein synthesis inhibition, reduction in oxidative stress, and prevention of DNA damage and cell death, by in vitro assay on renal cell lines. Therefore, it would be important in the future to assess whether L-tryptophan could also present these different actions. In addition, we cannot rule out the hypothesis that the L-tryptophan used here also prevented the effects of OTA on protein synthesis inhibition and contributed to the synthesis of IgA.

IgA is the main defense antibody in the GIT, having several protective functions, such as a prevention of environmental antigen influx into internal body compartments, neutralization of viruses, and microbial toxins [30].

To our knowledge, this is pioneering data to show the toxic effect of OTA on gut IgA and IgY and IgA^+^ cell levels and that treatment with L-tryptophan can neutralize these effects and improve the intestinal morphology in intoxicated chicks. Whether these findings have an impact on immune defense or animal performance will be investigated in the future.

## 4. Materials and Methods

### 4.1. Experimental Chicks and their Management

Thirty-six, one-day-old broiler chickens (Cobb 500) were used in the study, which were divided into four groups, and each group was placed in a separate cage, with a photoperiod of 12 h a day and a temperature of 30 °C in the first week and 28 °C in the second week of the experiment, under strict hygiene conditions. All birds had access to the same formulated feed, which was prepared according to Rostagno et al. [39] and water ad libitum, which was changed daily. Cages, feeders, and drinking fountains were also cleaned daily. Newly hatched chicks are more sensitive to mycotoxins, and in a previous investigation with 0.1, 0.7 and 1.4 mg OTA/kg, decreased leukocyte levels were detected only in a dose of 1.4 mg OTA/kg (data not shown). Therefore, one-day-old chicks and a high dose were chosen for this study. This dose is considered occasional but realistic (can be encountered occasionally during unfavorable weather conditions) according to Grenier et al. [24].

### 4.2. OTA and Tryptophan—Dose Preparation

The OTA (CAYM 11439; Cayman Chemical Company, Ann Arbor, MI, USA) was dissolved in absolute ethanol (concentration of 2 mg/mL) one day before starting the experiment. For inoculating, the OTA was diluted in sterile saline-phosphate buffer (PBS), and each animal was inoculated by gavage with 0.5 mL (1.4 mg/kg b.w.). The L-tryptophan (T0254; Sigma-Aldrich, San Louis, MO, USA) was resuspended in sterile PBS, and each animal was inoculated orally by gavage with 0.5 mL in a final concentration of 100 mg/kg b.w.

### 4.3. Experimental Design

The chickens were divided into four groups with nine animals each (negative control, OTA, OTA/L-tryptophan, and L-tryptophan control). On the first day of the experiment, the animals from the OTA and OTA/L-tryptophan groups were inoculated with 1.4 mg OTA/kg b.w. by gavage and the other groups were inoculated with PBS.

On the subsequent four days, the birds in the control group and the OTA group received a daily dose of sterile PBS and the birds in the L-tryptophan and OTA + L-tryptophan groups received a daily dose of L-tryptophan (100 mg/kg b.w.); all inoculations were administered orally by gavage. Fourteen days post OTA intoxication, the animals were euthanized by cervical dislocation, and the intestine fragments and fecal content were collected for processing. The experiment was conducted with the approval of the Ethics Committee on the Use of Animals at the State University of Londrina, PR, Brazil, approved on 11 December 2017 (CEUA No. 18289.2017.16), in accordance with the legislation and standards of the National Council of Animal Experimentation (CONCEA).

### 4.4. Histological and Immunohistochemistry Analysis

Duodenum fragments were fixed in 4% buffered formalin solution, and after 48 h, they were stored in 70% ethanol. The tissues were dehydrated in increasing concentrations of ethanol, diaphanized in absolute xylol, embedded in paraffin, and sectioned in a microtome with a thickness of 5 µm. Deparaffinized sections were stained with hematoxylin and eosin. The morphometry evaluation was performed by measuring the height of 30 villi and the depth of the adjacent crypts for each animal. For this, five animals from each group were randomly chosen. The program Motic Images Plus 2.0 (Motic Group Company, Hong Kong, China) was used to obtain images and perform measurements. For immunohistochemistry, the sections were incubated at 70 °C for 10 min for antigenic reactivation, which was followed by endogenous peroxidase blocking with 1% H_2_O_2_ for 1 h and with 1% bovine serum albumin (BSA) for three hours, both at room temperature (r.t.). This was incubated with anti-chicken IgA antibody conjugated with peroxidase (ab11/2817, Abcam Plc, Cambridge, UK) in dilutions of 1:100 for 1 h at r.t. and with substrate (diaminobenzidine—DAB 0.05% and H_2_O_2_ 0.3%) for 30 min; then, they were counterstained with Harris hematoxylin and analyzed under an optical microscope. The IgA^+^ cells were counted in 10 fields for each group (400× magnification).

### 4.5. Total IgY and IgY and IgA Levels in Fecal Content

Fecal content was collected from the cecum in 2 mL microtubes and stored in the −80 °C freezer until processing. To carry out the analyses, 0.250 g of the sample of each animal was diluted in 1 mL of 0.01 M PBS. The material was homogenized in a vortex and left to stand at 4 °C for 10 min before being centrifuged at 5590× *g* for 5 min. The supernatant was diluted in a proportion of 1:2 in PBS 0.01 M glycerol 40%, and the aliquots obtained were stored at −20 °C in a freezer until the ELISA was performed. To quantify IgA/IgY, an immunocapture ELISA was performed. The aliquots were thawed at r.t. and diluted in 1% PBS milk 1: 5000 for IgA and 1:100 for IgY analysis. High-affinity ELISA plates (655061. Greiner Bio-One, Frickenhausen, Germany) were sensitized with 0.25 µL of anti-chicken IgA capture antibody (A30103A. Bethyl Laboratories, Montgomery, TX, USA) and anti-chicken IgY capture antibody (A30104A. Bethyl Laboratories, Montgomery, TX, USA) and incubated at 4 °C overnight. Next, the plates were blocked with PBS milk 5% for 2 h at 25 °C and incubated with diluted samples and a standard curve for 1 h at 25 °C. Subsequently, the secondary antibodies anti-IgA (A30103P. Bethyl Laboratories, Montgomery, TX, USA) and anti-IgY (A9046, Sigma-Aldrich, San Louis, MI, USA) conjugated with peroxidase were added in dilutions of 1:25,000. The reaction was carried out with substrate (tetramethylbenzidine—TMB 0.01% and H_2_O_2_ 3%) for 15 min, followed by 2N H_2_SO_4_ solution. The plates were read in a spectrophotometer at 450 nm. To calculate the results, the optical densities obtained were compared to the standard curves of each plate. The standard curves were built using the CurveExpert 1.4 program (Hyams Development, Starkville, MS, USA, 2009).

### 4.6. Statistical Analysis

To verify the normality and homogeneity of the groups, the Lilliefors test and the Levene test were performed, respectively. Groups that did not show normality underwent statistical transformation and were subjected to one-way analysis of variance (ANOVA) to verify whether there was a difference between them, after which the Student–Newman–Keuls test was performed for multiple comparisons. It was not possible to normalize the depth of the crypts data, so the Kruskal–Wallis non-parametric test was used to analyze the differences between the groups and the Student–Newman–Keuls test was used for multiple comparisons. The data were considered significantly different when *p*-value < 0.05.

## 5. Conclusions

Acute intoxication with a single high OTA dose can be detrimental to intestinal health in chicks by changing the humoral immune response and intestinal morphology and, L-tryptophan treatment can mitigate these effects. On the basis of our preliminary findings, treatment with L-tryptophan could be a valid alternative means to reduce the harmful effects of OTA, which requires further study. The small sample size (*n* = 9) could be the limitation of this study because of the huge standard deviations of quantitative data shown in Figure 2, Figure 3 and Figure 5, which make it difficult to tell whether the significant difference really exist between OTA group and OTA-Tryptophan groups, particularly those in Figure 3.
